# Innovations in Injectables: An Expert Video Roundtable Discussion

**DOI:** 10.1093/asjof/ojae054

**Published:** 2024-07-22

**Authors:** Sachin M Shridharani, Jean Carruthers, Michael A C Kane, Lawrence Bass

The authors are pleased to present an expert roundtable discussion on innovations in injectables (Video). The discussants include a panel of leading experts in aesthetic surgery, and the video roundtable was recorded live at The Aesthetic Meeting 2024 in Vancouver, Canada. The panelists began with an overview of the history of neuromodulators and neurotoxins, summarizing Dr Carruthers’ pioneering work in treating glabellar lines, followed by an explanation of new and future applications of neuromodulators. They then discussed innovations in dermal fillers that have emerged in recent years that allow for more specialized applications in various treatments. The panelists all agreed that these developments have given them much greater flexibility in their practices for treating patients’ specific needs. The panelists concluded with a discussion of avoiding and managing adverse events in injection treatments, most importantly through correct technique and careful dosing, as well as appropriate drug treatments to counteract adverse events.

